# Midshaft clavicle fractures treatment: threaded Kirschner wire versus conservative approach

**DOI:** 10.1007/s11751-017-0293-7

**Published:** 2017-08-20

**Authors:** Valentino Coppa, Luca Dei Giudici, Stefano Cecconi, Mario Marinelli, Antonio Gigante

**Affiliations:** 1 Clinical Orthopaedics, Department of Clinical and Molecular Science, School of Medicine, Università Politecnica delle Marche, Via Tronto, 10/A, 60126 Ancona, Italy; 2 Clinic of Adult and Paediatric Orthopaedic, Azienda Ospedaliero-Universitaria, Ospedali Riuniti di Ancona, Ancona, Italy

**Keywords:** Clavicle fracture, Midshaft, Conservative treatment, Clavicle pinning, Mini invasive

## Abstract

Clavicle fractures are common, accounting for 2.6 to 10% of all fractures. Treatment of these fractures is usually non-surgical. Recent evidence, however, reveals that the final result of non-surgically midshaft clavicular fractures, particularly those with quite large displacements or shortening, is not like that which was previously thought. This study evaluated retrospectively all patients presented with a clavicle fracture at Emergency Department of our Institution, between January 2006 and December 2011. Fractures were classified according to Allman’s radiographic classification system, modified by Nordqvist and Petersson. Patients were distinguished into two groups: one that underwent conservative treatment with a “figure-of-8” orthosis and one that underwent surgery with reduction in fracture and fixation with intramedullary threaded Kirschner wire. Pin removal was performed after 4 weeks of rest in Gilchrist bandage, after clinical and radiographic evaluation demonstrating the bone healing. The QuickDASH score and the Constant Murley Shoulder Score were used to evaluate the clinical outcomes. The radiographic outcome was evaluated at 1 and 6 months of follow-up. Database review provided a final cohort of 58 patients, with similar demographic features. There was no significant difference in qDASH and CS between the two groups. The results of qDASH and CS evaluated in function of the radiographic outcome show a statistically significant correlation between the worst qDASH and CS results and the grade of malunion in both groups. In particular, we have found unsatisfactory results when final shortening of the clavicle was 20 mm or more. On radiographic evaluation, surgical treatment demonstrated a greater efficacy in reducing initial shortening of the fractured bone; this is in opposition to conservative treatment that results very often in malunion, shortening, anatomic alterations and loss of functionality. The use of intramedullary threaded Kirschner wire for fixation of midshaft clavicle fractures is a safe procedure and is recommended in case of shortening greater than 2 cm in high-function-demand patients.

## Introduction

Clavicle fractures are common lesions, ranging from 2.6 to 10% of all fractures and up to 44.1% of the fractures involving the upper girdle [1]. Males are generally more affected, with sport injuries representing the most described traumatic pattern in young patients, while falling on the ground is the most common in adults. A direct hit on the shoulder is the most common cause of midshaft clavicle fractures [2].

Traditionally, midshaft clavicular fractures have been managed non-operatively, even when substantially displaced [3, 4], with good to excellent results [5, 6]. Recent evidence, however, reveals that the final result of non-surgically midshaft clavicular fractures, particularly those with quite large displacements or shortening, is not like that which was previously thought, demonstrating higher rates of delayed union, non-union, shoulder weakness and residual pain [7, 8]. Several recent articles have characterized the symptoms reported with clavicular malunion, which is associated with substantial degrees of skeletal deformity, especially shortening of ≥2 cm [9].

Although many methods have been described for closed reduction in displaced clavicular shaft fractures, none has been consistently reliable in achieving and maintaining reduction. Thus, displaced midshaft fractures of the clavicle typically heal in approximately the same position as that seen on initial radiographs [9]. The limits associated with non-operative treatment are, in fact the risk of non-union, malunion, altered biomechanics of the upper girdle, deformity with unsatisfactory cosmetic results, and upper extremity weakness [7, 10–12]. These factors have caused an increase in the indications for surgical treatment [11, 13, 14].

Surgery finds absolute indication in the presence of open fractures, high comminution and dislocation of the fragments, high risk for in–out skin wounds, a shortening superior to 20 mm, floating shoulder and neurovascular lesions. Relative indications are polytraumas, painful malunions or non-unions [15, 16].

Operative treatment of displaced MSCFs can be achieved successfully using plates or intramedullary (IM) implants like Rush pins, Kirschner wires, or nails, but an optimal surgical technique is still not identified [4].

Aim of the present paper is to clinically evaluate the outcomes of two groups of patients suffering from displaced midshaft clavicle fracture, treated by conservative and by surgical treatment, depicting every possible association.

## Materials and methods

This study evaluated all patients presented at our Emergency Department with a clavicle fracture in a time frame ranging from 1 January 2006 to 31 December 2011.

Data about patients were gathered by retrospectively reviewing hospital records. Triage Informatic Records were reviewed first, filtering records for clavicle fracture only. The results of patients were evaluated according to the inclusion and exclusion criteria (shown below) to include those in the study. The enrolled patients were then reviewed on the Surgical Procedures Registry to divide surgical patients from non-surgical ones. Lastly, a double check was performed through the analysis of Radiological Imaging Database for a specific patient, from the time of admittance at the emergency department onward, to confirm surgical and non-surgical patients.

In regards to Institute’s Privacy Policy, it must be noted that every reported system and records provided were sorted by an anonymous identification number assigned at the admittance to the hospital.

Clavicle fractures were defined as displaced according to Postacchini et al. [17], therefore considering the distance between the inferior border of one bone fragment and that of the corresponding border of the other fragment at the fracture site if exceeding 3 mm on radiographs with a 1:1 magnification. Identified fracture was then classified according to Allman’s radiographic classification system [18], modified by Nordqvist and Petersson [1, 10, 19]. This system was chosen for its simplicity compared to other classification systems and for the proper prognostic predictively.

This classification sets 3 groups and 3 subgroups of fractures. Group I fractures include fractures of middle third. Group II and group III include fracture of lateral and medial third, respectively. Subgroups a, b, and c include undisplaced, displaced, and multifragmentary fracture, respectively [1].

We also divided the patients on the amount of the shortening evaluated at XR before and after treatment (group A: shortening less than 1 cm; group B: shortening more than 1 cm but less than 2 cm; and group C with shortening greater than 2 cm).

For every patient, a clinical assessment was performed, along with a standard biprojective XR of the affected shoulder girdle.

Patient’s inclusion criteria were fracture’s types IB and IC according to Nordqvist and Petersson, age ranging from 14 years old to 65 years old, a complete file record, the completion of the rehabilitation programme and the voluntary positively response to the last follow-up.

Exclusion criteria were fractures types II and III and IC with high comminution (more than four fragments), polytrauma, open or pathological fractures, infective or systemic disease, previous surgeries on the affected shoulder, floating shoulder, anatomical variations in respect of normal anatomy, previous traumas and previous rehabilitation treatments on the affected joint.

**Fig. 1:**
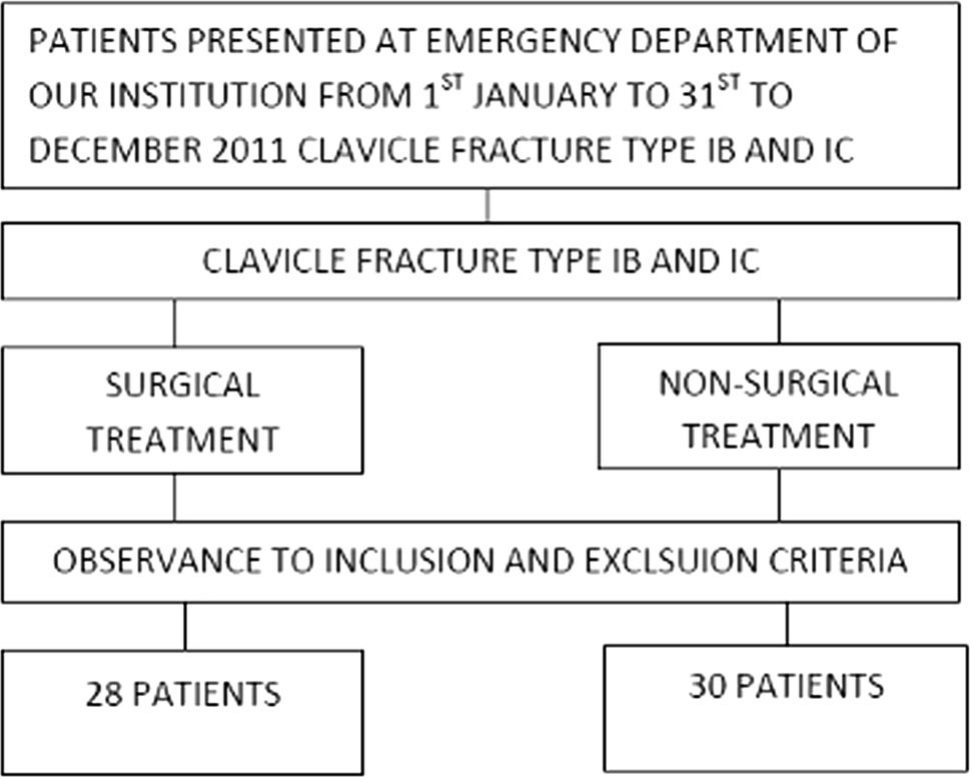
Algorithm used to determine the study cohort

Study cohort was determined according to the diagram provided in Fig. 1.

Concordance between observers, in regard of the treatment performed, was re-evaluated at the end of the study, obtaining a *K* coefficient >0.85.

### Non-operative treatment

Patients treated conservatively were managed with a “figure-of-8” orthosis. After its application, the patient underwent a plain radiograph to check the alignment of the fragments; patients were also instructed on its use, on how to maintain its correct position, how to re-tension and avoid axillary decubitus ulcers and compression of the neurovascular bundle.

All patients treated non-operatively have been invited, in the first phase, to avoid all active movements of shoulder and to perform slight mobilization movements of the hand and the elbow without load to prevent joint contractures and oedema.

After 4 weeks of treatment, patients were asked to remove the bandage and start to perform rehabilitation programme.

### Surgical technique

The patient candidates for surgical treatment were subjected to the reduction in fracture and fixation with intramedullary threaded Kirschner wire (K-wire) with a procedure similar to the Murray method [20].

**Fig. 2:**
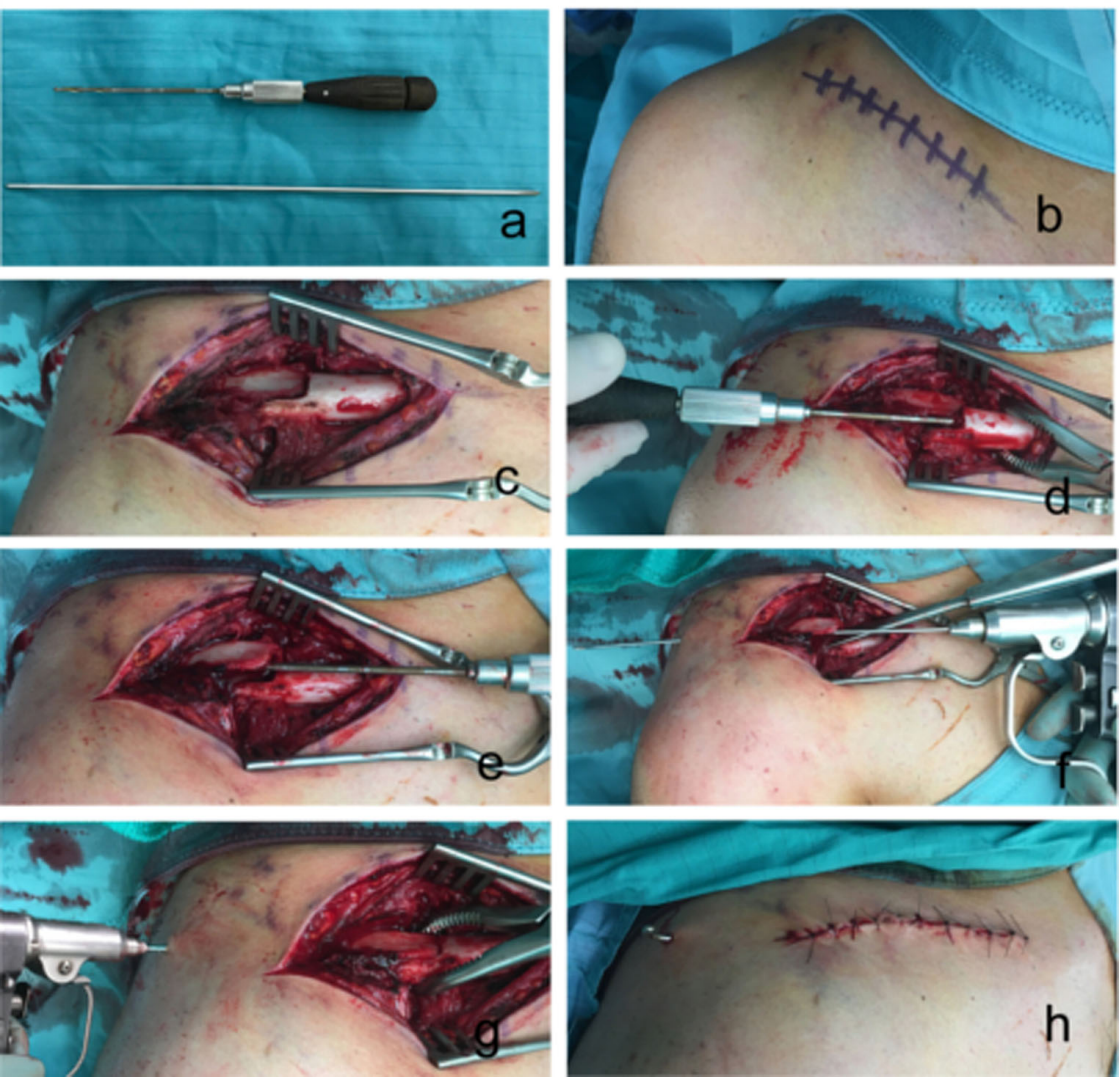
Figure shows the essential surgical instruments and the main steps of our technique

The patients underwent general anaesthesia. The technique (Fig. 2) provides the patient in the supine position on a radiolucent surgical table with a slight overflow of the arm out from the edge of the bed and with a slight inclination of the trunk to ensure freedom of movement of the arm.

A small incision (3–4 cm) (Fig. 2 b, c) was made at the level of the fracture in line with its major axis of the clavicle. After a blunt dissection of the soft tissue, the fracture site is reached. A 2.5-mm drilling bit is used to open the intramedullary canal (Fig. 2d, e). The threaded Kirschner wire (from 2 to 3 mm diameter according to the size of the intramedullary canal) was advanced in the lateral bone fragment intramedullary canal till the K-wire exits throughout the postero-lateral skin (Fig. 2f) and then (Fig. 2g) it is advanced in the medial bone fragment intramedullary canal (in–out technique). The advancement of the K-wire was controlled with an image intensifier.

To provide the best reduction, the bone nearby the fracture site was gently deperiosted, facilitating the alignment of the bone ends. A cerclage with a high strength non-absorbable suture #2 (FiberWire^®^, Arthrex, Naples U.S.) was performed in the presence of a third fragment, or multifragmentary fracture.

**Fig. 3:**
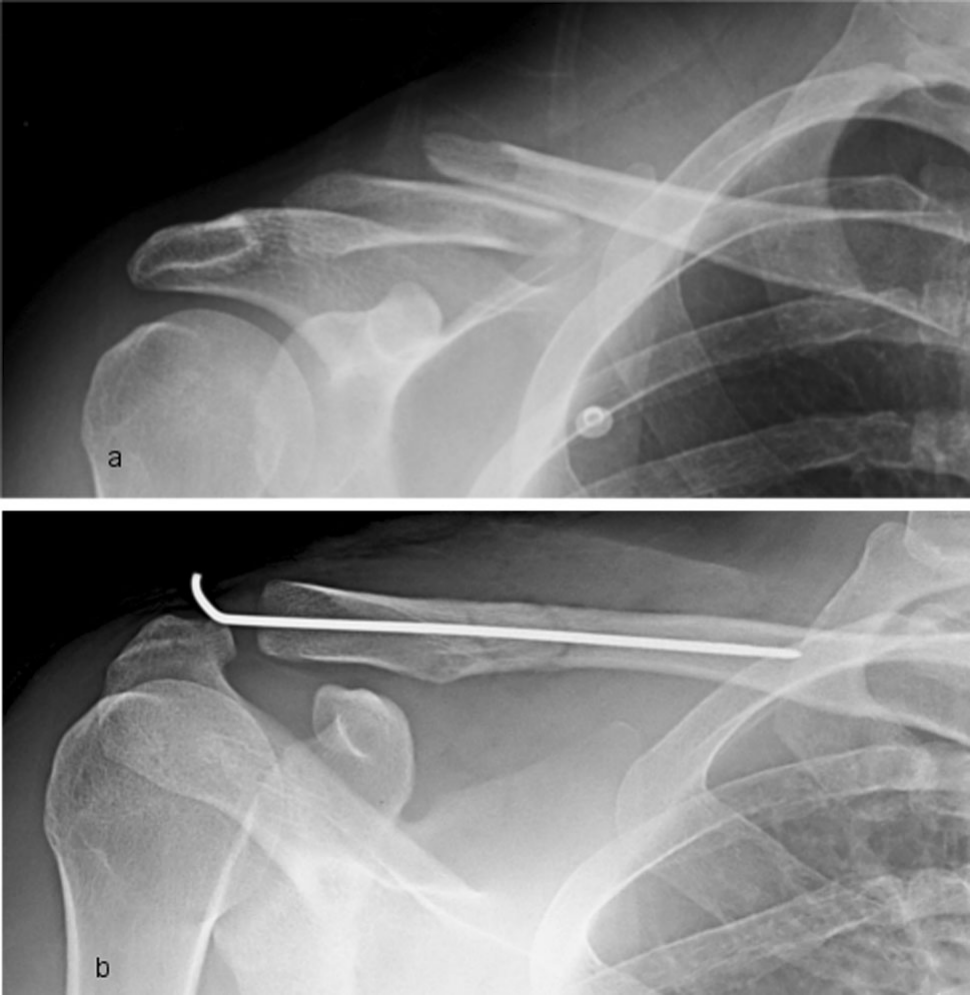
Figure shows the radiography of midshaft clavicle fracture **a** before and **b** after the surgical treatment

At the end of the procedure, the K-wire folded and cut close to the exit point in order to minimize irritation of the soft tissues, but leaving a sufficient part for an easier removal afterwards (Fig. 2h). A control radiography was performed at the end of the procedure (Fig. 3). A Gilchrist bandage was applied for 4 weeks postoperatively. Acetaminophen use was suggested in case of persistent pain, usually in the first postoperative days. Pin removal was allowed after 4 weeks of rest in an outpatient setting without any anaesthesia.

### Outcome measure

The Quick Disabilities of the Arm, Shoulder and Hand (qDASH) score [21, 22] score and the Constant Murley Shoulder (CS) score [23] were used to evaluate the clinical outcomes. The minimal detectable change at the 95% confidence level (MDC95%) ranged from 16 to 20 QuickDASH points (with a mean of 18) [22]. The MIC 95% limit of CS was found at a mean change of 24 points [24].

The radiographic outcome was determined by a pre-treatment, post-treatment and at 1 and 6 months of follow-up standard posteroanterior thorax radiograph according to Smekal et al. [25]. Post-treatment radiographs were firstly evaluated for the presence of delayed union, non-union and malunion. Union was defined as bony bridging over the fracture gap [26]. The malunion is evaluated by calculating the results in shortening in centimetres compared to the contralateral side. The images were all analysed using an open-source software (OsiriX, v5.0.2) in terms of mean displacement and shortening.

### Statistical analysis

The two groups have been compared with the Fisher’s exact test to confirm the homogeneity about age, gender, side affected, fracture type according to Allman’s Classification modified by Nordqvist and Petersson [18, 19] and displacement grade of the fracture before treatment.

The results of the qDASH and CS were analysed in function of the treatment performed (surgical or non-surgical) by the Wilcoxon test.

In patients who have undergone surgery, the results of qDASH and CS were analysed in function of radiographic outcome by Wilcoxon test. In patients treated non-operatively, the same evaluation has been performed by Kruskall–Wallis test. Post-hoc power analysis on the two groups constituting the cohort has been performed.

The radiographic outcomes were evaluated in function of the treatment by the Student’s t test.

## Results

Database review provided a total of 243 patients with clavicular fractures of which 174 were excluded because they matched one of the exclusion criteria and 11 were eligible to be included in the study but lost to follow-up (dropouts). Of these, 4 were unreachable, 3 were off site and 4 have refused to be included in the study for personal reasons.

A final cohort of 58 patients (51 males, 7 females; man age 38.35 years old; median 35.64 years old) who met the inclusion and exclusion criteria was enrolled to constitute two groups: one of 30 patients that underwent conservative treatment and one of 28 patients that underwent surgical treatment. Out of these, we found a total of 25 fractures on the right side and 33 on the left side. The two groups were significantly homogeneous concerning age, gender, side, fracture subtype, and displacement extent before treatment (analysed by Fisher’s exact test, respectively, with *p* = 1, *p* = 0.595, *p* = 0.706, *p* = 0.802, Table 1).

**Table 1:** Demographic data, fracture classification and initial clavicle shortening

	Surgical group	Non-surgical group
Total	28	30
Age (years)	39.5 ± 15.7	37.4 ± 15.5
Sex		
Male	26 (92.9%)	25 (83.3%)
Female	2 (7.1%)	5 (16.7%)
Affected side		
Right	12 (42.9%)	13 (43.3%)
Left	16 (57.1%)	17 (56.7%)
Fracture classification		
1b	9 (32.1%)	10 (33.3%)
1c	19 (67.9%)	20 (66.7%)
Initial shortening		
*a* (<1 cm)	9 (32.1%)	10 (33.33%)
*b* (1/2 cm)	10 (35.7%)	10 (33.33%)
*c* (>2 cm)	9 (32.1%)	10 (33.33%)
Follow-up (years)	4 ± 1.9	3.8 ± 1.5

According to Nordqvist and Petersson classification, a total of 19 fractures were classified as type IB and 39 as type IC [1, 10, 18, 19].

Functional outcomes were analysed at a mean follow-up of 48 months (range 28.32–74.52 months) for the surgical-treated group and 45 months (range 22.68–73.92 months) for the non-surgical-treated group.

### Operative versus non-operative group

**Fig. 4:**
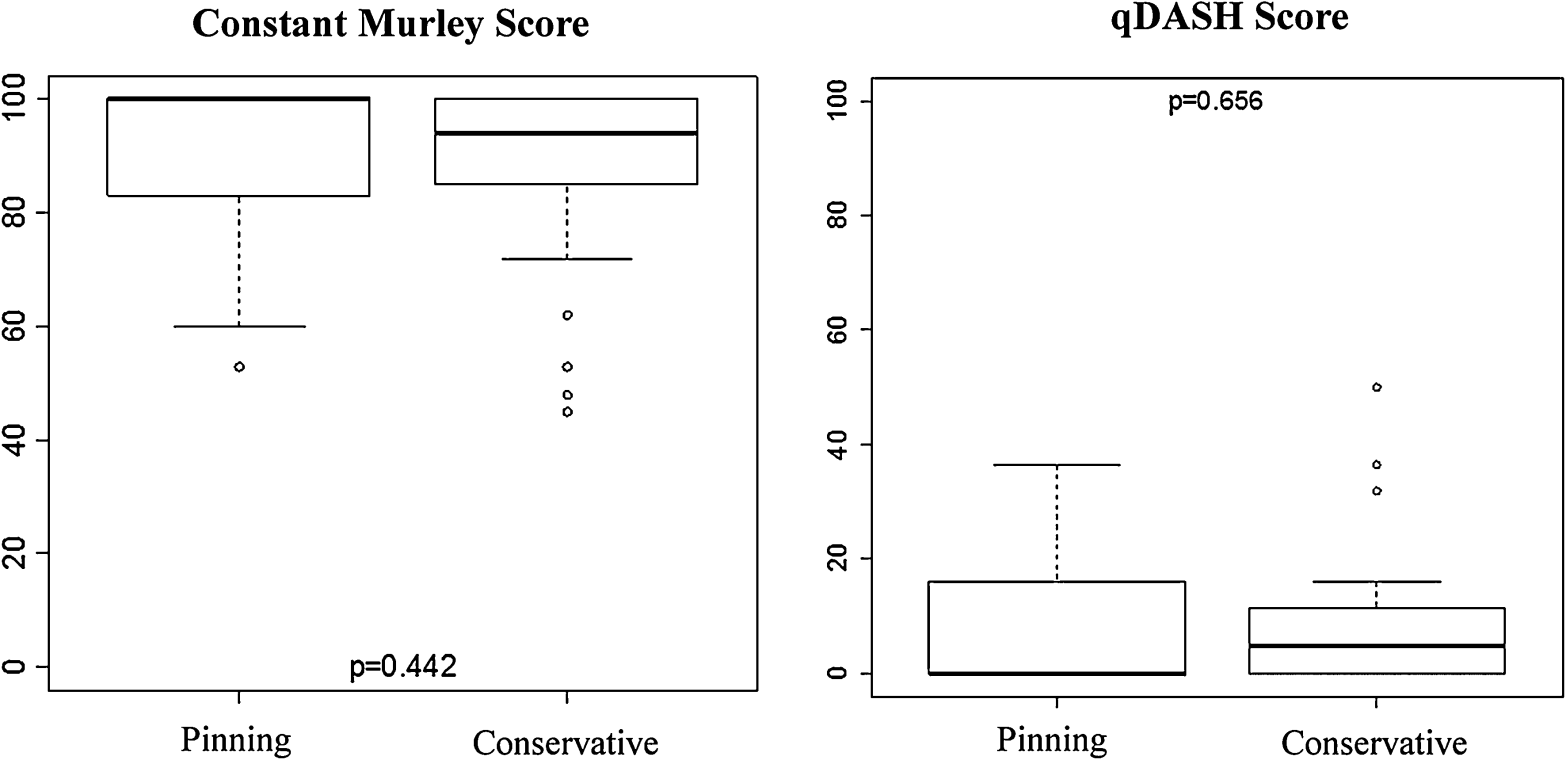
*Box plot* with outcomes of **a** constant score (best score = 0), **b** qDASH score (best score = 0) compared by the type of treatment

The results of qDASH and CS obtained after treatment have showed no statistically significant difference between the two groups (Fig. 4a, b).

### Clinical outcome in function of radiographic outcome

**Fig. 5:**
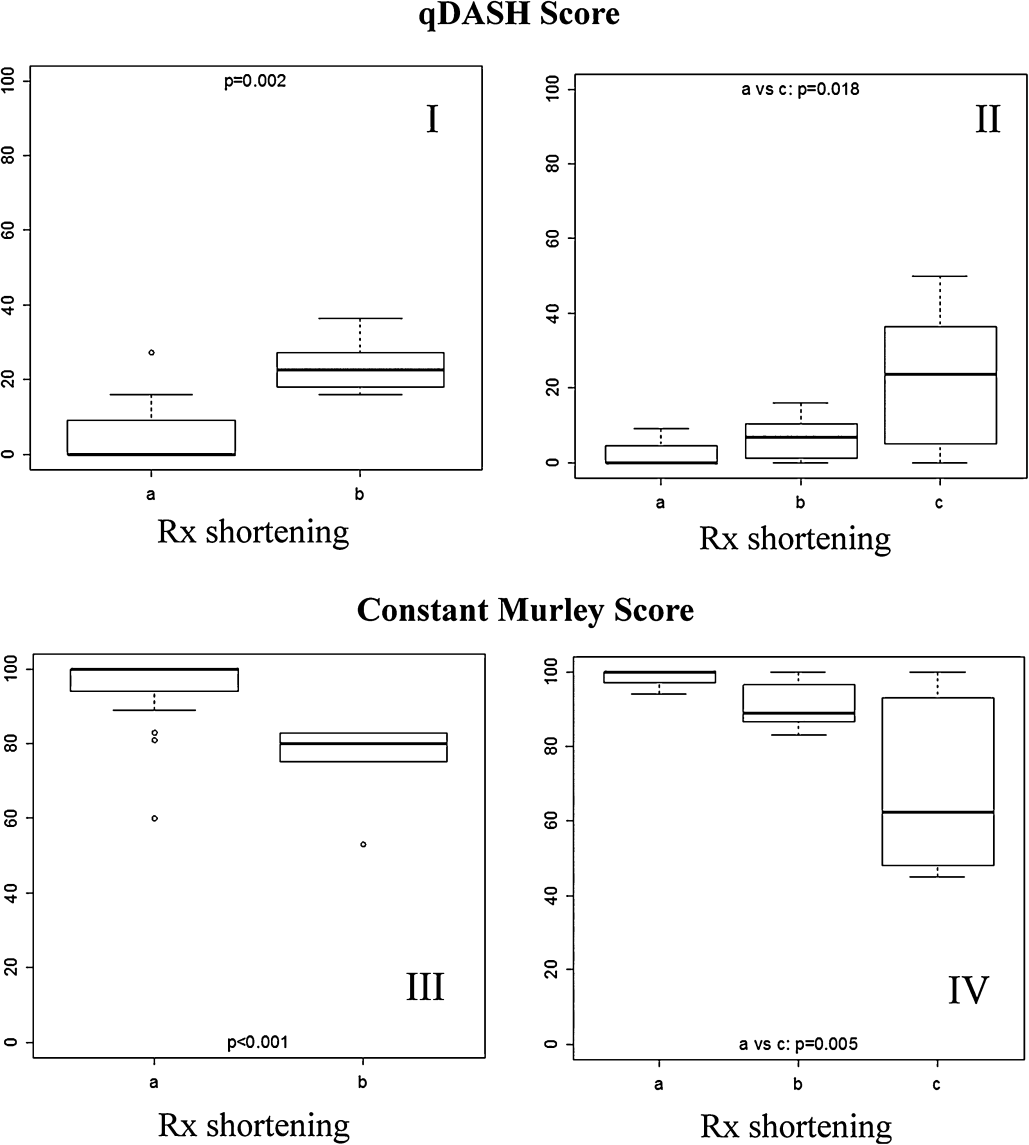
*Box plot* of DASH (*I*, *II*) score and constant score divided (*III*, *IV*) by treatment (*I*, *III* surgical treatment; *II*, *IV* conservative treatment and radiograph shortening

The results of qDASH evaluated in function of the radiographic outcome (Fig. 5I, II) showed a statistically significant correlation between the worst (lower) qDASH results and the grade of radiograph shortening in both groups (respectively, Wilcoxon test *p* = 0.002 in surgically treated group and Kruskall–Wallis test *p* = 0.018 in non-operative treated group).

In particular, in the group non-operatively treated the difference between the medians of the results of the qDASH of patients with shortening <2 cm and those with shortening greater than 2 cm was approximately 25 points.

In the group surgically treated, instead, there is a difference of about 20 points in the median of the results of the qDASH of patients without shortening and those with shortening <2 cm.

The results of CS evaluated in function of the radiographic outcome (Fig. 5III, IV) have shown a statistically significant correlation between the worst CS results and the grade of shortening in both groups (respectively, Wilcoxon test *p* < 0.001 in surgically treated group and test Kruskall-Wallis *p* = 0.005 in non-operatively treated group).

In the group surgically treated, there is a difference of about 20 points between the medians of the CS results of patients without shortening (median of 100 points) and those with shortening of less than 2 cm (median of 80 points). In the group treated non-operatively, the difference between the medians of the CS results of patients with shortening of less than 2 cm and those with shortening greater than 2 cm is about 30 points (in patients without shortening the median is 100 points, in those with shortening of less than 2 cm the median is 90 points in the patients with shortening greater than 2 cm the median is 60 points).

### Radiographic outcome

In the group of patients surgically treated, a reduction in displacement was observed in 25 (89.29%) cases and not in 3 (10.71%) cases. The improvement was statistically significant (*p* = 0.032). In particular, there were no patients with a shortening greater than 2 cm at the end of treatment.

In non-operatively treated group, the improvement in radiographic outcome was not statistically significant (*p* = 0.464) with 23 cases (76.67%) in which there was no improvement of the shortening and only 6 cases (20.69%) in which was obtained an improvement of the fracture displacement by the figure-of-8 orthosis application. One case (3.33%) showed a worse radiographic outcome than pre-treatment.

### Complications

No iatrogenic lesions have been reported after both treatment options. No implant failure occurred. Superficial infection at the site of the surgical approach for the fracture reduction was seen in 1 patient (3.57%) with hypertrophic scar formation. Three patients (10.71%) of the surgical groups have complained to feel pain in case of touch at the level of the previous fracture zone.

We found one (3.33%) non-union in the non-operative-treated group and none in the surgical-treated group.

## Discussion

Aim of our study was to evaluate, on a mid- to long-term follow-up, two different treatments for midshaft clavicular fractures, underlining any possible difference in terms of functional and radiographic outcomes, and non-union rates.

Our results showed that there are no significant differences between qDASH and CS in the two groups at the latest follow-up. We hypothesize this is due to the fact that not all types of clavicle fractures require surgical treatment and have a good outcome both with a surgical treatment and a conservative one. This is confirmed by the study of Nordqvist and Petersson [10].

In this study, of 225 patients with midshaft clavicle fracture treated conservatively, 185 were asymptomatic. Canadian Orthopaedic Trauma Society (COTS) (2007) [27] reported better results, evaluated by DASH score, in favour of the surgical group at all-time points. The magnitude of the difference, however, was less than 10 points, which is not considered a clinically relevant difference [21, 28].

A particular correlation was found statistically and clinically significant between the functional outcomes and the entity of displacement observed at follow-up, with worst and lower scores associated to a greater shortening. In those patients, surgical treatment with elastic stable intramedullary nailing (ESIN) performed significantly better compared to conservative treatment [29]. This is supported by the results of Ledger et al. [12] and Ristevski et al. [30].

They have shown in their patients that an average shortening, respectively, of 21.4 mm and 21.1 mm, is associated with an alteration of the normal anatomy of the scapular girdle with a worsening of shoulder function. Our results are in line with those of Hill et al. [7] who reported unsatisfactory results in 31% of patients when the final shortening of the clavicle was 20 mm or more. Lazarides et al. [31] showed similar results: out of all evaluated patients in their study (132), 34 (25.8%) were dissatisfied. They found that the increase in the shortening associated with accumulation and final clavicular shortening of more than 18 mm in male patients, and of more than 14 mm in female patients, was significantly associated with an unsatisfactory result.

Oroko et al. [32] found 3 of 41 patients with shortening of 15 mm or more who had low Constant disability scores, but this could be attributed to other factors. Smekal et al. [26] evaluated ESIN versus non-operative with randomized, controlled, clinical trial. These Authors showed a significant positive correlation between DASH score at endpoint and definite shortening, and between patient satisfaction and definite shortening. Furthermore, patients suffering from sequelae after 2 years had an average shortening of 6.1% (65.2%).

Rasmussen et al. [33], however, advocate conservative treatment of midshaft clavicle fractures with a shortening of 20 mm or more. They have clinically evaluated 130 patients treated conservatively. They have found patients with a shortening less than 20 mm had a mean difference in the Constant–Murley Score of 7.2. Mean difference between the two groups was 0.7 with no correlation between shortening of the clavicle and the clinical outcome. They have concluded a shortening of more than 20 mm was not associated with a poorer clinical outcome.

Nordqvist et al. [10] have found that comminuted fractures (1C) do not behave significantly worse than do non-comminuted fractures (1B) and they have suggested that selection for surgical treatment cannot be based on the appearance of the fracture. However, Nowak et al. have shown that comminution in clavicle fractures is a negative prognostic indicator [14]. O’Neill et al. [1] in their series have found 6.2% of displaced simple fractures (1B) and 11.2% of comminuted fractures reach out to non-union.

On radiographic evaluation, surgical treatment demonstrated a great efficacy in reducing initial shortening of the fractured bone; this is in opposition to conservative treatment that, according also to other results showed in the literature, results very often in malunion, shortening, anatomic alterations and loss of functionality [7, 12, 31, 34].

Moreover, the vast majority of our surgically treated patients has reported a faster recovery, with a mean time of 3 months to return to full activities, while the non-surgical patients needed a mean time of 6 months; this results is in accordance with the data shown by Naveen et al. [35].

Non-union incidence in our study was 3.33% in the non-surgical-threaded group and is lower than was stated in the literature for the type of fractures studied that is usually over 10% [7, 26–28]. Our opinion about it is that in our study are excluded patients with high fracture comminution and other factors which are associated with clavicular non-union. In literature, indeed, it is reported that factors which predisposed to non-union include open fractures, refractures, associated multiple injuries, significant displacement, high comminution and inadequate immobilization [13] and it is suggested by several Authors [14, 15, 35] that patients with non-union risk factors aforementioned, in particular high comminution, should be surgically treated by open reduction and internal fixation (ORIF) by plating.

Among complications of surgical treatment, the most common one associated with nailing is the medial migration with skin irritation [36–38]. None of our patients reported such complication. This could be explained by the usage of a threaded Kirschner wire that has its medial extremity threaded that can provide higher stability of the construct, especially to telescopic forces. This finding is supported by results provided by Frigg et al. [39] that showed a reduction in medial migration when using an end cap for titanium elastic nail (TEN).

Biomechanically, plate fixation is superior to intramedullary fixation because it better resists the bending and torsional forces that occur during elevation of the upper extremity above shoulder level [40]. Patients treated with plate fixation can be allowed full range of motion once their soft tissues have healed. Disadvantages of plate fixation include the slightly higher infection rates, soft tissue irritation due to plate decubitus, and the risk of refracture after plate removal [8, 27].

The patients undergoing plating the implant removal need another surgery done under general anaesthesia, with a large-sized incision, while the intramedullary devices can be removed as outpatients with or without local anaesthesia [8]. In our experience, to obtain an anatomic reduction of clavicular fracture and application of treated k-wire, in particular in case of severe displacement of the fragments, is required an exposure similar to ORIF by plating (Fig. 2c–g) but the removal of threaded k-wire is easier than plate and it was performed in outpatient setting without any anaesthesia in all cases.

This paper presents some limitations. In first place, it is a retrospective analysis, without treatment randomization, limiting its power; this study design also presents only one follow-up at 46 months. This could obviously lead to a loss of information about trends and minor details that could have arose during the interval between treatment and follow-up, but one of the strongest points of the study groups is the completeness of the records that therefore limits the bias due to the design to a minimum, giving a precise picture of the outcome of the patient. A minor limitation could be the number of patients analysed, but even if other papers evaluated bigger groups, the statistical analysis performed allowed to state that the 58 patients included represent an adequate cohort in order to obtain statistical significance. Another bias could be the radiographic methods to evaluate the clavicle shortening. In fact, some recent studies have shown that plain radiograph-based measurements of midshaft clavicle shortening are precise, but inaccurate [41] and there is only a low correlation between X-ray and CT measurement [42]. However, Smekal et al. [25] have demonstrated that it is possible to obtain a result comparable to CT images with a posteroanterior thorax radiograph. Furthermore, it is our opinion, always in agreement with the same Authors, that the CT should not be used to evaluate an acute fracture since the supine position may change the fracture displacement and shortening.

In conclusion, with this study, we can assert that patients who would benefit more from an intramedullary synthesis in case of midshaft fracture of the clavicle are patients with a high degree of initial displacement, particularly if shortening greater than 2 cm.

We also noticed that when surgical treatment is performed, to achieve the best clinical results, it is necessary to obtain an anatomic reduction, in particular in relation to the length.

Surgeon should also investigate the real patient’s needs, in particular in workers and sportsman, as the surgical treatment is able to ensure a return to complete activity of the upper limb more quickly.

The patient’s profile related to fracture is, in our opinion, a new way to look after the surgical indications: the fracture characteristic in itself should not be the only landmark, but understanding the patient’s abilities and demands should rule the surgeon decision-making.
